# Who Smokes in Europe? Data From 12 European Countries in the TackSHS Survey (2017–2018)

**DOI:** 10.2188/jea.JE20190344

**Published:** 2021-02-05

**Authors:** Silvano Gallus, Alessandra Lugo, Xiaoqiu Liu, Panagiotis Behrakis, Roberto Boffi, Cristina Bosetti, Giulia Carreras, Liliane Chatenoud, Luke Clancy, Xavier Continente, Ruaraidh Dobson, Tobias Effertz, Filippos T. Filippidis, Marcela Fu, Gergana Geshanova, Giuseppe Gorini, Sheila Keogan, Hristo Ivanov, María J. Lopez, Angel Lopez-Nicolas, José Precioso, Krzysztof Przewozniak, Cornel Radu-Loghin, Ario Ruprecht, Sean Semple, Joan B. Soriano, Polina Starchenko, Marta Trapero-Bertran, Olena Tigova, Anna S. Tzortzi, Constantine Vardavas, Vergina K. Vyzikidou, Paolo Colombo, Esteve Fernandez, the TackSHS Project Investigators

**Affiliations:** 1 Department of Environmental Health Sciences, Istituto di Ricerche Farmacologiche Mario Negri IRCCS, Milan, Italy; 2 Institute of Public Health of the American College of Greece, Athens, Greece; 3 Fondazione IRCCS Istituto Nazionale dei Tumori, Milan, Italy; 4 Department of Oncology, Istituto di Ricerche Farmacologiche Mario Negri IRCCS, Milan, Italy; 5 Istituto per lo Studio, la Prevenzione e la Rete Oncologica, Florence, Italy; 6 Department of Public Health, Istituto di Ricerche Farmacologiche Mario Negri IRCCS, Milan, Italy; 7 TobaccoFree Research Institute Ireland, Dublin, Ireland; 8 Agència de Salut Pública de Barcelona, Barcelona, Spain; 9 CIBER en Epidemiología y Salud Pública (CIBERESP) (Biomedical Research Centre Network for Epidemiology and Public Health), Madrid, Spain; 10 Institut d’investigació Biomèdica Sant Pau (IIB St. Pau), Barcelona, Spain; 11 University of Stirling, Stirling, Scotland; 12 University of Hamburg, Hamburg Business School, Institute for Law & Economics, Hamburg, Germany; 13 Department of Primary Care and Public Health, Imperial College, London, UK; 14 Tobacco Control Unit, Institut Català d’Oncologia, L’Hospitalet de Llobregat, Barcelona, Spain; 15 Tobacco Control Research Unit, Institut d’Investigació Biomèdica de Bellvitge, L’Hospitalet de Llobregat, Barcelona, Spain; 16 Universitat de Barcelona, L’Hospitalet de Llobregat, Barcelona, Spain; 17 Consortium for Biomedical Research in Respiratory Diseases (CIBER en Enfermedades Respiratorias, CIBERES), Madrid, Spain; 18 Smoke Free Life Coalition, Sofia, Bulgaria; 19 Universidad Politécnica de Cartagena, Cartagena, Spain; 20 Instituto de Educação, Universidade do Minho, Braga, Portugal; 21 Maria Sklodowska-Curie National Research Institute of Oncology, Warsaw, Poland; 22 Foundation “Smart Health - Health in 3D”, Warsaw, Poland; 23 Collegium Civitas, Warsaw, Poland; 24 European Network for Smoking Prevention, Bruxelles, Belgium; 25 Hospital Universitario La Princesa, Madrid, Spain; 26 Research Institute for Evaluation and Public Policies (IRAPP), Universitat Internacional de Catalunya (UIC), Barcelona, Spain; 27 DOXA Institute, Milan, Italy

**Keywords:** tobacco, cigarette smoking, smoking prevalence, cross-sectional study, survey, Europe, TackSHS

## Abstract

**Background:**

Population data on tobacco use and its determinants require continuous monitoring and careful inter-country comparison. We aimed to provide the most up-to-date estimates on tobacco smoking from a large cross-sectional survey, conducted in selected European countries.

**Methods:**

Within the TackSHS Project, a face-to-face survey on smoking was conducted in 2017–2018 in 12 countries: Bulgaria, England, France, Germany, Greece, Ireland, Italy, Latvia, Poland, Portugal, Romania, and Spain, representing around 80% of the 432 million European Union (EU) adult population. In each country, a representative sample of around 1,000 subjects aged 15 years and older was interviewed, for a total of 11,902 participants.

**Results:**

Overall, 25.9% of participants were current smokers (31.0% of men and 21.2% of women, *P* < 0.001), while 16.5% were former smokers. Smoking prevalence ranged from 18.9% in Italy to 37.0% in Bulgaria. It decreased with increasing age (compared to <45, multivariable odds ratio [OR] for ≥65 year, 0.31; 95% confidence interval [CI], 0.27–0.36), level of education (OR for low vs high, 1.32; 95% CI, 1.17–1.48) and self-rated household economic level (OR for low vs high, 2.05; 95% CI, 1.74–2.42). The same patterns were found in both sexes.

**Conclusions:**

These smoking prevalence estimates represent the most up-to-date evidence in Europe. From them, it can be derived that there are more than 112 million current smokers in the EU-28. Lower socio-economic status is a major determinant of smoking habit in both sexes.

## INTRODUCTION

In 2008, the World Health Organization (WHO) introduced the MPOWER policy package, a series of technical measures and resources to assist country-level implementation of the WHO Framework Convention on Tobacco Control (FCTC), developed in response to the globalization of the tobacco epidemic. The first of these technical measures involves the monitoring of prevention policies and tobacco use.^1^

Several national surveillance systems are available to monitor smoking prevalence and trends in most countries.^2^ However, data from country-specific populations are poorly comparable, since they are not collected using a standardized study design—including sampling methods, mode of interviewing and questionnaires—which represents a prerequisite for valid comparisons of smoking prevalence and smoking patterns across different countries.^3^^,^^4^

To the best of our knowledge, the only standardized and representative investigations that systematically monitor smoking prevalence among adults in more than one European country, are the WHO Global Adult Tobacco Survey (GATS)^5^^,^^6^; the Survey of Health, Ageing and Retirement in Europe (SHARE)^7^; and the European Commission (EC) Special Eurobarometers.^8^^–^^10^ GATS covers three European Union (EU) member states (MS; ie, Greece, Poland, and Romania)^5^^,^^6^; SHARE is a longitudinal study, conducted in five waves between 2004 and 2013, collecting information on smoking habit among individuals aged 50 years and older from 20 European countries^7^; Eurobarometer is a representative survey conducted in all the 28 EU MS on approximately 1,000 face-to-face interviews per country, based on selected thematic studies. The most recent Eurobarometer surveys focusing on tobacco smoking indicated that the overall smoking prevalence in the EU had slightly decreased from 29% in 2009 to 26% in 2014, and remained stable until 2017.^8^^,^^9^^,^^11^ Among the other tobacco control projects, the International Tobacco Control Policy Evaluation Project (the ITC Project) includes longitudinal studies in 10 European countries. However, all these studies, which include samples not representative of the general population or smokers only, cannot provide smoking prevalence estimates.^12^ The above-mentioned European surveillance systems are important and useful tools; however, they are not always able to investigate in-depth key tobacco control measures and smoking determinants. Consequently, a few specific studies, mainly supported by the EC, have been conducted to analyse this issue.^3^^,^^13^^–^^15^ These include, the Pricing Policies and Control of Tobacco in Europe (PPACTE) survey, conducted in 2010 to investigate the effectiveness of pricing policies on tobacco control in Europe^3^^,^^16^^,^^17^; and the present survey, conducted within the TackSHS Project (http://tackshs.eu),^18^ aimed to improve understanding of exposure to second-hand tobacco smoke (SHS) and e-cigarette aerosol in Europe.

In this paper we aimed to describe the TackSHS survey, a multi-country survey conducted in 2017–2018 in 12 EU MS, providing the main results on smoking prevalence and its correlates in Europe.

## METHODS

The TackSHS survey^18^ has been conducted in 12 strategically selected EU countries (Bulgaria, England, France, Germany, Greece, Ireland, Italy, Latvia, Poland, Portugal, Romania, and Spain), representing geographical, legislative and cultural variations across the EU.

This cross-sectional study was coordinated by Istituto di Ricerche Farmacologiche Mario Negri IRCCS (Mario Negri Institute; Milan, Italy). The fieldwork was conducted by DOXA, the Italian branch of the Worldwide Independent Network/Gallup International Association, and its European partners.

### Sample selection

In each country, we considered a sample of around 1,000 individuals aged 15 years and older (participants’ age was ≥16 in England, ≥18 in Ireland, 15–64 in Greece, and 15–74 years in Latvia), representative of the general population in terms of age, sex, habitat (ie, geographic area and/or size of municipality) and, in some countries, socio-economic characteristics. The survey included a total of 11,902 subjects. The whole population domain represented 79.2% of the whole EU population aged 15 years or over (432 million inhabitants).

Sampling methods varied across countries: in Bulgaria, Greece, Italy, Latvia and Romania, a multi-stage sampling was used and respondents were randomly chosen to be representative of the population in terms of sex, age, and geographic area (in Italy, representativeness by socio-economic characteristics was also ensured); in Germany, Ireland, Poland, Portugal, and Spain, stratified random sampling was used, combining also quotas on sex and age and social class in Ireland; in England, United Kingdom, cluster sampling with quotas on age, sex, socio-economic status (SES), region, urban/rural dwelling was used to ensure national representativeness; in France, quotas on age, sex, region, and city size were used for the selection of survey participants.

### Questionnaire

A first draft of the survey questionnaire was developed in English language by the researchers from Mario Negri Institute, based on previous tobacco use and secondhand smoke exposure questionnaires.^3^^,^^19^^–^^21^ A specific commission with six experts among project partners was created to review the questionnaire and to produce a second version. The final version of the English questionnaire was then developed through the collaboration with all the partners of the TackSHS consortium (eMaterial 1). The questionnaire was translated into Italian language by Mario Negri Institute’s researchers, and into Bulgarian, French, German, Greek, Latvian, Russian, Polish, Portuguese, Romanian, and Spanish by DOXA partners. The translated versions of the questionnaire were revised and validated by bilingual (ie, local language and English language) tobacco control experts.

The questionnaire contains four sections: socio-economic and demographic characteristics; cigarette smoking habit and e-cigarette use; exposure to secondhand smoke (SHS) and e-cigarette aerosol, plus cigarette smoking and e-cigarette use, in different indoor and outdoor places; and attitudes and perceptions on smoke-free regulations and awareness of SHS harmful effects.

### Fieldwork

Ad hoc trained interviewers conducted a face-to-face, computer-assisted personal interviewing (CAPI) survey in all the 12 European countries. A test fieldwork was conducted by DOXA in Italy in November 2016 among 1,059 participants. The fieldwork in the other 11 countries was conducted between June 2017 (in Romania) and October 2018 (in Latvia). Table 1 provides information on survey characteristics for each country, including fieldwork period, sample size, age range and sampling methods.

**Table 1.** Information about survey methods and sample characteristics in 12 European countries. TackSHS Project, 2017–2018

Country	Fieldwork dates	Sample size^a^	Age range (years)	Survey mode	Sampling method	Representativeness
Bulgaria	Oct 2017	1,050	≥15	CAPI	Multi-stage stratified random sampling with quota support	Age, sex, habitat
England	Jan–Feb 2018	1,013	≥16	CAPI	Cluster sampling with quota support	Age, sex, habitat, SES
France	Nov–Dec 2017	1,018	≥15	CAPI	Quota method	Age, sex, habitat
Germany	Jun 2018	1,031	≥15	CAPI	Stratified random sampling	Age, sex, habitat
Greece	Jun–Jul 2018	1,000	15–64	CAPI	Multi-stage random sampling	Age, sex, habitat
Ireland	Nov 2017	941	≥18	CAPI	Stratified random sampling with quota support	Age, sex, habitat, SES
Italy	Nov 2016	1,059	≥15	CAPI	Multi-stage random sampling	Age, sex, habitat, SES
Latvia	Oct 2018	1,022	15–74	CAPI	Multi-stage random sampling	Age, sex, habitat
Poland	Sep 2018	724	≥16	CAPI	Multi-stage stratified random sampling with cluster support	Age, sex, habitat
Portugal	Nov–Dec 2017	1,000	≥15	CAPI	Stratified sampling	Age, sex, habitat
Romania	Jun–Jul 2017	1,018	≥15	CAPI	Multi-stage stratified random sampling with quota support	Age, sex, habitat, SES
Spain	Oct 2017	1,026	≥15	CAPI	Stratified random sampling	Age, sex, habitat

Total	Jun 2017–Oct 2018^b^	11,902	≥15	CAPI	—	

CAPI, computer assisted personal interview; SES, socio-economic status.

^a^Raw sample size.

^b^This period does not include the time of the survey conducted in Italy (ie, Nov–Dec 2016) since Italy was the pilot country. The participants from Italy are included in all the analyses.

### Variables and definitions

Demographic characteristics included age (categorized as <25, 25–44, 45–64, ≥65 years) and sex (men and women). Level of education was categorized as country-specific tertiles of schooling years. Self-assessment of household family economic status relative to the country-specific population was classified into three levels (higher than average, average, and lower than average). Never smokers were defined as participants who had never smoked or had smoked less than 100 cigarettes in their lifetime. Smokers were defined as participants who reported smoking at least 100 cigarettes (including roll-your-own cigarettes) during their lifetime. Current smokers were smokers who reported smoking at the time they participated in this survey, while ex-smokers were smokers who stopped smoking by the time they participated in this survey.^3^^,^^21^ Smokers also provided information on age of starting smoking and the number of cigarettes smoked per day. For each country, we calculated the male-to-female smoking prevalence ratio as the current smoking prevalence in men divided by that in women. The 12 countries were classified into Northern (England, Ireland, and Latvia), Western (France and Germany), Southern (Italy, Greece, Portugal, and Spain), and Eastern (Bulgaria, Poland, and Romania), following United Nation M49 standard,^22^ and halved by their gross domestic product (GDP) per capita^23^ into <25.000 € (Latvia, Romania, Poland, Portugal, Greece, and Bulgaria) and ≥25.000 € (England, France, Germany, Ireland, Italy, and Spain).

### Ethical issues

We obtained study approval from a local Ethics Committee in each of the 12 countries (eTable 1). Details on the survey characteristics were provided to all participants by suitably qualified professionals through a structured information sheet, and all the participants provided their consents by ticking the electronic field in the CAPI questionnaire. The study protocol has been registered in ClinicalTrials.gov (ID: NCT02928536).

The procedures for recruitment of subjects, data collection, storage, and protection (based on anonymous identification code) are in accordance with the current country-specific legislation. This was ratified and signed by DOXA and each of its European partners.

### Statistical analysis

Statistical weights were used to generate representative estimates of the general population of each country (individual weight). To calculate results for the entire sample, we applied “country weights”, which combined individual weights with an additional weighting factor, with each country contributing in proportion to its population aged 15 years or over, obtained by Eurostat.^24^

We considered descriptive statistics including relative frequency (%) and its corresponding 95% confidence intervals (CIs) for categorical variables, and mean and standard deviation (SD) for continuous variables.

ORs and their corresponding 95% CIs were estimated using multilevel logistic random-effects models, to take into account the heterogeneity between the 12 European countries. The study country effects were considered as random intercepts, and sex, age, and level of education as adjusting variables. Country weights were used in all logistic regression models. Analyses were performed with SAS 9.4 (SAS Institute; Cary, NC, USA).

## RESULTS

Among 11,902 participants, 57.6% (95% CI, 56.8–58.5%) described themselves as never smokers (49.5% in men and 65.1% in women), 16.5% (95% CI, 15.8–17.2%) as ex-smokers (19.5% in men and 13.7% in women) and 25.9% (95% CI, 25.1–26.7%) as current smokers (31.0% in men and 21.2% in women; Table 2). The smoking prevalence was below 20% in Italy (18.9%), Ireland (19.6%), and England (19.8%), and over 35% in Bulgaria (37.0%) and Portugal (36.8%). Smoking prevalence in men ranged from 20.3% in England to 42.0% in Portugal, and in women from 15.3% in Germany to 34.4% in Bulgaria. Average smoking intensity was 14.7 cigarettes per day (SD, 8.8), ranging from 12.4 (SD, 7.5) in Italy to 17.1 (SD, 15.8) in Greece.

**Table 2.** Country-specific prevalence of never, ex- and current smokers, and smoking intensity in the European population aged ≥15 years. TackSHS Project, 2017–2018

Country	Prevalence (%)						Number of cigarettes per day,^a^ mean (SD)		

	Never smokers	Ex-smokers	Current smokers						

			Total	Men	Women	M/F ratio	Total	Men	Women
Bulgaria	46.4	16.6	37.0	39.8	34.4	1.16	14.9 (7.9)	17.1 (8.2)	12.5 (6.8)
England	59.9	20.2	19.8	20.3	19.5	1.04	15.5 (9.4)	15.0 (9.4)	15.8 (9.4)
France	48.8	20.1	31.0	36.7	25.9	1.42	13.6 (8.7)	15.4 (9.0)	11.3 (7.7)
Germany	63.4	12.9	23.7	32.4	15.3	2.12	15.9 (7.0)	17.1 (7.0)	13.4 (6.4)
Greece	40.8	25.4	33.8	35.0	32.6	1.07	17.1 (15.8)	21.1 (18.9)	12.9 (10.1)
Ireland	65.4	15.0	19.6	22.1	17.2	1.28	NA		
Italy	69.8	11.3	18.9	22.6	15.5	1.46	12.4 (7.5)	12.9 (7.9)	11.7 (7.1)
Latvia	55.7	16.3	28.1	41.3	16.0	2.58	12.9 (7.0)	13.9 (6.8)	10.6 (7.0)
Poland	62.9	13.5	23.6	28.7	19.4	1.48	16.4 (11.2)	18.7 (12.7)	13.6 (8.5)
Portugal	48.5	14.7	36.8	42.0	32.1	1.31	14.1 (9.4)	16.2 (10.9)	11.7 (6.4)
Romania	47.4	18.7	34.0	38.7	29.5	1.31	16.2 (10.0)	18.4 (10.4)	13.3 (8.9)
Spain	47.8	20.4	31.8	37.3	26.6	1.40	14.0 (8.0)	14.5 (8.7)	13.3 (7.0)

Total^b^	57.6	16.5	25.9	31.0	21.2	1.46	14.7 (8.8)	16.0 (9.4)	13.0 (7.7)

M/F, male to female; SD, standard deviation.

^a^Smoking intensity, including both manufactured and roll-your-own cigarettes among current smokers (men and women combined). Questions on cigarette smoking intensity was not asked in Ireland. In the other 11 countries, 2,938 out of 3,050 current smokers provided information on the number of cigarettes smoked per day.

^b^Country weights were applied, which combined individual weights with an additional weighting factor, with each country contributing in proportion to its population aged 15 years or over, obtained by Eurostat.^24^

Among current smokers, 76.1% started smoking before age 18 years and 96.3% before 25 years. Mean age at starting regular smoking was 17.4 (SD, 4.5) overall, 17.1 (SD, 4.1) in males, and 17.8 (SD, 4.8) years in female smokers, and varied between 15.2 (SD, 4.2) in England and 19.1 years (SD, 4.2) in Romania (data not shown in tables).

Table 3 provides the ORs for current versus non-smokers according to selected individual-level and country-specific characteristics. Smoking prevalence was higher in men than in women (OR 1.67; 95% CI, 1.53–1.82). Compared with subjects aged <45 years, ORs were 0.97 (95% CI, 0.89–1.07) for 45–64 years, and 0.31 (95% CI, 0.27–0.36) for ≥65 years. Compared with high level of education, the ORs were 1.39 (95% CI, 1.25–1.55) for medium and 1.32 (95% CI, 1.17–1.48) for low level of education (*P* for trend <0.001). Compared to those with a high household economic status, the ORs were 1.33 (95% CI, 1.15–1.54) and 2.05 (95% CI, 1.74–2.42) for those with an average and low economic status, respectively. The patterns observed for all the individual-level characteristics were consistent in men and women. Compared with Northern Europe, the ORs of smoking were 1.58 (95% CI, 0.98–2.56) in Western, 1.52 (95% CI, 0.93–2.49) in Southern and 1.57 (95% CI, 0.92–2.70) in Eastern European countries. Compared to Northern Europe, the OR in men was higher in Western Europe (OR 2.17; 95% CI, 1.35–3.48), Southern Europe (OR 1.77; 95% CI, 1.09–2.87) and Eastern Europe (OR 1.87; 95% CI, 1.10–3.18). Smoking prevalence showed no significant difference between countries with lower and higher GDP per capita, the ORs for less wealthy countries being 1.22 (95% CI, 0.82–1.81).

**Table 3.** OR for current smokers vs non-smokers (never and ex-smokers combined) and corresponding 95% CI in the European population aged ≥15 years, overall and by sex, according to selected individual-level and country-specific characteristics.^a^ TackSHS Project, 2017–2018

Characteristics	*N*^b^	Current smoking					

		Total		Men		Women	

		%	OR (95% CI)^c^	%	OR (95% CI)^c^	%	OR (95% CI)^c^
Individual level characteristics							
Sex							
Women	6,270	21.2	1^d^				
Men	5,632	31.0	**1.67 (1.53–1.82)**				
Age group, years							
<25	1,446	24.5	1^d^	29.4	1^d^	19.1	1^d^
25–44	4,079	31.1		36.6		26.0	
45–64	4,330	28.9	0.97 (0.89–1.07)	33.3	0.93 (0.82–1.05)	24.9	1.03 (0.90–1.17)
≥65	2,047	11.8	**0.31 (0.27–0.36)**	16.8	**0.36 (0.29–0.43)**	7.8	**0.26 (0.21–0.33)**
*P* for trend			**<0.001**		**<0.001**		**<0.001**
Level of education^e^							
High	3,241	24.4	1^d^	27.4	1^d^	21.5	1^d^
Medium	4,172	29.4	**1.39 (1.25–1.55)**	36.0	**1.56 (1.35–1.82)**	23.2	**1.23 (1.05–1.45)**
Low	4,486	23.6	**1.32 (1.17–1.48)**	28.7	**1.38 (1.18–1.61)**	19.2	**1.25 (1.06–1.48)**
*P* for trend			**<0.001**		**<0.001**		**0.012**
Household economic status^f^							
Higher than average	1,588	20.9	1^d^	**23.3**	1^d^	**18.4**	1^d^
Average	6,264	25.9	**1.33 (1.15–1.54)**	**29.6**	**1.38 (1.13–1.69)**	**22.4**	**1.29 (1.05–1.60)**
Lower than average	2,905	31.5	**2.05 (1.74–2.42)**	**38.8**	**2.27 (1.80–2.85)**	**26.0**	**1.90 (1.50–2.41)**
*P* for trend			**<0.001**		**<0.001**		**<0.001**
Country-specific characteristics							
Geographic area							
Northern Europe	2,976	20.1	1^d^	21.1	1^d^	19.2	1^d^
Western Europe	2,049	26.9	1.58 (0.98–2.56)	34.3	**2.17 (1.35–3.48)**	20.0	1.13 (0.66–1.94)
Southern Europe	4,085	26.2	1.52 (0.93–2.49)	30.5	**1.77 (1.09–2.87)**	22.2	1.35 (0.78–2.34)
Eastern Europe	2,792	28.2	1.57 (0.92–2.70)	33.1	**1.87 (1.10–3.18)**	24.0	1.37 (0.75–2.50)
GDP per capita							
>25.000 €	6,088	24.7	1^d^	30.0	1^d^	19.9	1^d^
≤25.000 €	5,814	29.9	1.22 (0.82–1.81)	34.6	1.16 (0.73–1.85)	25.8	1.30 (0.87–1.95)

CI, confidence interval; GDP, gross domestic product; OR, odds ratio.

^a^Country weights were applied, which combined individual weights with an additional weighting factor, with each country contributing in proportion to its population aged 15 years or over, obtained by Eurostat.^24^

^b^Raw sample size.

^c^ORs and their corresponding 95% CIs were estimated using multilevel logistic random-effects models, to take into account the heterogeneity between the 12 European countries. The study country effects were considered as random intercepts, and sex, age and level of education as adjusting variables. Estimates in bold are statistically significant at 0.05 level.

^d^Reference category.

^e^Level of education was categorized as country-specific tertiles of schooling years. The sum does not add to the total because of a few missing values.

^f^Self-assessment of household (family) economic status relative to the country-specific population. This variable is missing for all German subjects, 79 Latvian and, 35 Romanian subjects.

## DISCUSSION

Approximately one out of four adults is a current smoker in Europe, including one out of three men and one out of five women. Smoking prevalence showed large differences across European countries, being highest in Eastern Europe (28%) and lowest in Northern Europe (20%).

In both men and women, smoking prevalence is highest among young adults aged 25–44 years. Almost all current smokers in Europe started smoking before aged 25 years. Therefore, this study confirms that teenagers and young adults are the most vulnerable group to start smoking^3^ and thus are the most important target population for tobacco control interventions aimed to decrease nicotine addiction initiation.

Smoking prevalence among women, exceeding 15% in all the 12 countries, is in an advanced stage of the tobacco epidemic.^3^^,^^25^ However, notable differences were observed in the male-to-female smoking prevalence ratio, ranging from 1.04 in England to 2.58 in Latvia. This suggests that some countries (likely those with a higher male-to-female smoking prevalence ratio) are in an earlier phase compared with others.^3^^,^^25^

Multilevel logistic analyses indicate that the level of education was inversely related to smoking prevalence in both men and women. Analysing other individual level and country specific indicators of SES, we also found that smoking prevalence was systematically higher in the sub-population with low SES. This finding was independent of sex, age, and education, as reported in a few other investigations.^26^ Our results are in broad agreement with current evidence,^27^^,^^28^ and underline the need for specific measures specifically focused on low socio-economic groups to reduce health inequalities from tobacco.

In general, our findings are consistent with those reported in the Global Health Observatory data repository of the WHO^29^ and those found in the 2017 Eurobarometer survey.^8^ The latter study shows a smoking prevalence for the EU of 26% overall, 30% in men, and 20% in women, thus very similar to our estimates. In Italy, the smoking prevalence found in TackSHS survey (19% overall, 23% in men, and 16% in women) appears slightly lower than that found in the 2016 national survey conducted on around 3,000 subjects (ie, 22% overall, 27% in men, and 17% in women).^30^ Some major discrepancies with current evidence from the scientific literature were noted particularly in Portugal, where we found a smoking prevalence greater than 30% for both men and women. Our estimates are in fact substantially higher than those reported before 2015 for daily smoking (lower than 20%).^2^^,^^31^^,^^32^ The different definition of current smoking and the different sampling methodology used are not sufficient to explain the substantial gap found in smoking prevalence. Moreover, the distribution of our Portuguese sample by sex and age group is very similar to the official distribution provided by Eurostat.^24^ Other adult smoking prevalence estimates for Portugal, including those from the 2010 PPACTE study (32% overall, 36% in men, and 29% in women),^3^ or the 2015 WHO estimates (32% in men and 14% in women),^29^ or the 2017 Eurobarometer survey (26% overall)^8^ are more similar—but still substantially lower—than our estimates.

Overall, the smoking prevalence remained almost the same (from 27.2% to 26.4%) in the 11 countries that were included in both the TackSHS and PPACTE (2010) surveys, using similar methodologies and definitions. For France, Portugal, Romania, and Spain, we observed increases in smoking prevalence over the last decade but a decrease from 36% to 20% in Ireland.

The trends in smoking prevalence found in our data match those from the Eurobarometer surveys: the comparison between the 2017^8^ and 2009^11^ surveys indicated a decrease in smoking prevalence for all countries except for France and Portugal. Smoking prevalence estimates in Europe remain substantially higher as compared to those from other high-income countries, such as the United States, Canada, and Australia, where less than 15% of adults are current smokers.^2^^,^^33^

The limitations of the present study include some differences of sampling in the study countries. Notwithstanding, all samples selected were representative of their population in terms of age, sex, and geographic area and/or size of municipality. The age range of the participants was slightly different in some countries. In particular, in Greece the sample was limited to subjects aged 15–64 years, thus likely providing an over-estimation of the adult smoking prevalence. However, even assuming that the smoking prevalence in the elderly (representing in Greece 25% of the adult population^24^) was 50% that in younger adults—as observed in the other 11 countries—we would estimate a smoking prevalence around 30%, still similar to the provided estimate.

The strengths of our survey include the representativeness of the adult population of the 12 selected European countries, the use of the same questionnaire in the 12 countries sampled, developed by a group of experts on tobacco control, the standardized use of a single definition of current smokers, and the use of face-to-face interviews.

Our data indicate that there are 112 million current smokers in the EU, including 65 million men and 47 million women. We confirm that the large majority of smokers started smoking when they were adolescents, and that lower SES is a major determinant of smoking habit in Europe. Tobacco control measures to decrease smoking initiation and to promote smoking cessation should, therefore, be targeted at young people and the sub-group with lower SES, respectively. Increasing tobacco prices, through the adoption of additional excise taxes, has been shown to be more effective among younger generations and in economically deprived populations.^28^^,^^34^ Therefore, price increase is still likely to be a particularly effective tobacco control strategy, particularly in those European countries where tobacco price is still relatively low and resources for tobacco control are scarce.^35^

## APPENDIX A. SUPPLEMENTARY DATA

eMaterial 1. Survey questionnaire

eTable 1. Approvals of study protocol from Ethics Committees in 12 European countries. TackSHS Project 2017–2018.

## References

[1] WHO. WHO Report on the Global Tobacco Epidemic, 2008: The MPOWER package. Geneva, World Health Organization. Available online at https://www.who.int/tobacco/mpower/mpower_report_full_2008.pdf (last access: 10 July 2019). 2008.

[2] GBD 2015 Tobacco Collaborators Smoking prevalence and attributable disease burden in 195 countries and territories, 1990–2015: a systematic analysis from the Global Burden of Disease Study 2015. Lancet. 2017;389(10082):1885–1906. 10.1016/S0140-6736(17)30819-X 28390697 PMC5439023

[3] Gallus S, Lugo A, La Vecchia C, Pricing Policies And Control of Tobacco in Europe (PPACTE) project: cross-national comparison of smoking prevalence in 18 European countries. Eur J Cancer Prev. 2014;23(3):177–185. 10.1097/CEJ.0000000000000009 24441832

[4] Bogdanovica I, Godfrey F, McNeill A, Britton J Smoking prevalence in the European Union: a comparison of national and transnational prevalence survey methods and results. Tob Control. 2011;20(1):e4. 10.1136/tc.2010.036103 20966129 PMC3003865

[5] Centers for Disease Control and Prevention. Global Adult Tobacco Survey Collaborative Group. *Global Adult Tobacco Survey (GATS): Core Questionnaire with Optional Questions, Version 2.0*. Atlanta, GA.

[6] Mbulo L, Palipudi KM, Nelson-Blutcher G, Murty KS, Asma S; Global Adult Tobacco Survey Collaborative Group The process of cessation among current tobacco smokers: a cross-sectional data analysis from 21 countries, global adult tobacco survey, 2009–2013. Prev Chronic Dis. 2015;12:E151. 10.5888/pcd12.150146 26378897 PMC4576423

[7] Serrano-Alarcón M, Kunst AE, Bosdriesz JR, Perelman J Tobacco control policies and smoking among older adults: a longitudinal analysis of 10 European countries. Addiction. 2019;114(6):1076–1085. 10.1111/add.14577 30868688 PMC6593806

[8] Special Eurobarometer 458 report. Attitudes of Europeans towards tobacco and electronic cigarettes. 2017.

[9] Special Eurobarometer 429 report. Attitudes of Europeans towards tobacco and electronic cigarettes. 2015.

[10] Special Eurobarometer 485 report. Attitudes of Europeans towards tobacco. 2012.

[11] Special Eurobarometer 332 report. Tobacco. 2010.

[12] ITC Project. International Tobacco Control Policy Evaluation Project. Available online at https://itcproject.org/ (last accessed on: 08 January 2020).

[13] Balia S Survival expectations, subjective health and smoking: evidence from SHARE. Empir Econ. 2014;47(2):753–780. 10.1007/s00181-013-0750-1

[14] Trias-Llimós S, Muszyńska MM, Cámara AD, Janssen F Smoking cessation among European older adults: the contributions of marital and employment transitions by gender. Eur J Ageing. 2017;14(2):189–198. 10.1007/s10433-016-0401-4 28579935 PMC5435786

[15] ECRHS. European Community Respiratory Health Survey. Available online at http://www.ecrhs.org/Default.htm (last access: 10 July 2019).

[16] Gallus S, Lugo A, Ghislandi S, La Vecchia C, Gilmore AB Roll-your-own cigarettes in Europe: use, weight and implications for fiscal policies. Eur J Cancer Prev. 2014;23(3):186–192. 10.1097/CEJ.0000000000000010 24500021 PMC4127802

[17] Joossens L, Lugo A, La Vecchia C, Gilmore AB, Clancy L, Gallus S Illicit cigarettes and hand-rolled tobacco in 18 European countries: a cross-sectional survey. Tob Control. 2014;23(e1):e17–e23. 10.1136/tobaccocontrol-2012-050644 23233420 PMC3812425

[18] Fernández E, López MJ, Gallus S, ; TackSHS Project Investigators; TackSHS Project Investigators Tackling second-hand exposure to tobacco smoke and aerosols of electronic cigarettes: the TackSHS project protocol. Gac Sanit. 2020;34(1):77–82. 10.1016/j.gaceta.2019.07.002 31558386

[19] Pérez-Ríos M, Schiaffino A, López MJ, Questionnaire-based second-hand smoke assessment in adults. Eur J Public Health. 2013;23(5):763–767. 10.1093/eurpub/cks069 22683770

[20] Sureda X, Martínez-Sánchez JM, Fu M, Impact of the Spanish smoke-free legislation on adult, non-smoker exposure to secondhand smoke: cross-sectional surveys before (2004) and after (2012) legislation. PLoS One. 2014;9(2):e89430. 10.1371/journal.pone.0089430 24586774 PMC3937341

[21] IARC. IARC Handbooks of Cancer Prevention Volume 12. Methods for Evaluating Tobacco Control Policies. Available online at https://www.iarc.fr/wp-content/uploads/2018/07/Tobacco_vol12.pdf (last accessed on: 1 August 2019). 2008.

[22] Statistics Division. Department of Economic and Social Affairs. United Nations Standard country or area codes for statistical use (M49). 1999.

[23] World Bank. GDP per capita (current US$), World Bank national accounts data and OECD National Accounts data files. Available online at https://data.worldbank.org/indicator/NY.GDP.PCAP.CD?locations=EU (last accessed on: 1 August 2019). 2018.

[24] European Commission. Eurostat. Database. Available online at https://ec.europa.eu/eurostat/data/database (last access: 10 July 2019).

[25] Lopez AD, Collishaw NE, Piha T A descriptive model of the cigarette epidemic in developed countries. Tob Control. 1994;3(3):242–247. 10.1136/tc.3.3.242

[26] Huisman M, Kunst AE, Mackenbach JP Inequalities in the prevalence of smoking in the European Union: comparing education and income. Prev Med. 2005;40(6):756–764. 10.1016/j.ypmed.2004.09.022 15850876

[27] Bosdriesz JR, Willemsen MC, Stronks K, Kunst AE Tobacco control policy and socio-economic inequalities in smoking in 27 European countries. Drug Alcohol Depend. 2016;165:79–86. 10.1016/j.drugalcdep.2016.05.020 27262899

[28] Hiscock R, Bauld L, Amos A, Fidler JA, Munafò M Socioeconomic status and smoking: a review. Ann N Y Acad Sci. 2012;1248:107–123. 10.1111/j.1749-6632.2011.06202.x 22092035

[29] WHO. Tobacco use. Data by country. Global Health Observatory data repository. Available online at http://apps.who.int/gho/data/node.main.65 (last accessed on: 10 December 2019). 2015.

[30] Lugo A, Zuccaro P, Pacifici R, Smoking in Italy in 2015–2016: prevalence, trends, roll-your-own cigarettes, and attitudes towards incoming regulations. Tumori. 2017;103(4):353–359. 10.5301/tj.5000644 28574129

[31] Carreira H, Pereira M, Azevedo A, Lunet N Trends in the prevalence of smoking in Portugal: a systematic review. BMC Public Health. 2012;12:958. 10.1186/1471-2458-12-958 23137286 PMC3544737

[32] Drope J, Schluger N, Cahn Z, et al. *The Tobacco Atlas*. Atlanta: American Cancer Society and Vital Strategies. 2018.

[33] CDC. Centers for Disease Control and Prevention. Current Cigarette Smoking Among Adults in the United States. Available online at https://www.cdc.gov/tobacco/data_statistics/fact_sheets/adult_data/cig_smoking/index.htm (last access: 10 July 2019). 2019.

[34] IARC. *IARC Handbooks of Cancer Prevention. Vol. 14. Effectiveness of Tax and Price Policies for Tobacco Control*. Lyon, France; 2011. 10.1136/tc.2010.039982 21115556

[35] Joossens L, Feliu A, Fernandez E. The Tobacco Control Scale 2019 in Europe. Brussels: Association of European Cancer Leagues, Catalan Institute of Oncology. Available from: http://www.tobaccocontrolscale.org/TCS2019.pdf (last access: 30 June 2020). 2020.

