# Supplementary material for: Who Smokes in Europe? Data From 12 European Countries in the TackSHS Survey (2017–2018)
Source: J Epidemiol. 2021 Feb 5;31(2):145–51. doi: 10.2188/jea.JE20190344 (PMC7813769; doi:10.2188/jea.JE20190344)
Supplement: Supplementary file 1 [file je-31-145-s001.pdf]

eMaterial 1. Survey questionnaire

SURVEY QUESTIONNAIRE

SECTION A. Socio-economic and demographic characteristics

A0. The interviewer confirms that the participant information sheet has been read and delivered to the survey participant, and that the survey participant agrees to participate in the study |_|

A1.Age (years) |_|_|

A2.Sex |_| (M = Male, F = Female)

A3.Country of birth:

A4.Highest education level attended

Schooling (years) |_|_|

Level of education

no school	1
primary	2
lower secondary	3
upper secondary school	4
university.	5

A5.Geographic area (Nielsen region)

A6.Marital status

Married/live-in partner	1
Divorced/separated	2
Widowed	3
Single	4

A7.Self-assessment of the household (family) economic status relative to the country-specific population (5 levels)

Much higher than average in your country	1
Quite higher than average	2
On average in your country	3
Quite lower than average	4
Much lower than average	5

A8.Profession

Entrepreneur, Professional (self-employed)	1
Executive, Director, Top Management (employed)	2
Middle Manager (supervisor, high level technician)	3
Teacher (employed)	4
White-Collar (employed)	5
Owner of shop, Artisan (self-employed)	6
Blue-Collar skilled	7

Blue-Collar unskilled	8
Farmer (self-employed)	9
Rural worker (employed)	10
Housewife (working only in the home)	11
Retired	12
Unemployed (or looking for first job)	13
Student	14
Other, namely: ___________________________	15

A9. Your height |_|_|_| cm

A10. Your weight |_|_|_| Kg

SECTION B. Cigarette smoking habit and e-cigarette use

[To all]

B1. Have you smoked at least 100 cigarettes (including hand-rolled cigarettes) in your entire life?
→ No 1
→ Yes 2

[To ever smokers - code 2 at B1]

B2. Do you currently smoke cigarettes (including hand-rolled cigarettes)?
→ No 1
→ Yes 2

[To ex-smokers only - code 1 at B2]

B3. How long has it been since you last smoked cigarettes?
→ Within the past month (less than 1 month ago) 1
→ Within the past 3 months (1 month but less than 3 months ago) 2
→ Within the past 6 months (3 month but less than 6 months ago) 3
→ Within the past year (6 month but less than 1 year ago) 4
→ More than 1 year; 5 n° of years: |_|_|

[To smokers and ex-smokers only - code 2 at B1]

B4. Do (or did) you smoke cigarettes (including hand-rolled cigarettes) on a daily basis, or less than daily?
→ Daily 1
Manufactured cigarettes/day |_|_|
Hand-rolled cigarettes/day |_|_|
→ Less than daily 2
Manufactured cigarettes/week |_|_|
Hand-rolled cigarettes/week |_|_|
[if none, write 00; if he/she does not answer, write 99]

(To smokers and ex smokers only - code 2 at B1)

B5. How old were you when you first started smoking? |_|_|
[if he/she does not answer, write 99]

[To all]

B6. Have you ever heard about electronic cigarettes (e-cigarette)? Do you use or did you use it:
→ I have never heard about e-cigarette 1
→ I heard about it but I have never used it 2
→ I've just tried it a couple of times 3
→ I used it in the past (not over the last 30 days) 4
→ I use it occasionally (5 days or less in the last 30 days) 5
→ I use it regularly (more than 5 days in the last 30 days) 6

[To past or current users of e-cig - code 4-6 at B6]

B7. Which of the following sentences better describes the consequences of e-cigarette use on your CURRENT cigarette smoking behaviour?

→ I did not smoke cigarettes before, and I still do not smoke	1
→ I quit smoking	2
→ I substantially decreased the number of cigarettes per day	3
→ I slightly decreased the number of cigarettes per day	4
→ I did not modify my smoking habit	5
→ I increased the number of cigarettes per day	6
→ I started again (relapsed) to smoke cigarettes	7
→ I have started to smoke cigarettes	8

[To past or current users of e-cig - code 4-6 at B6]

B8. On average, how many puffs per day do (or did) you approximately make?

n° of puffs per day: |_|_|_|

[if participant is indecisive, ask to provide an approximate number of puffs; missing=999; less than 1 puff per day=998; more than 1000 puffs per day=997]

[To past or current users of e-cig - code 4-6 at B6]

B9. Which type of e-cigarette liquid do (or did) you more frequently use?

→ with nicotine (with or without flavours)	1
→ only vapour (no nicotine)	2
→ vapour and flavours (no nicotine)	3

[To past or current users of e-cig - code 4-6 at B6]

B10. Which type of e-cigarette device do (or did) you more frequently use?

→ Rechargeable	1
→ Disposable	2
→ Mods and variable voltage devices	3

[ONLY IN COUNTRIES WHERE IT IS ON SALE (e.g., Italy and UK); to all]

B11. Have you ever heard about heat-not-burn tobacco products? Do you use or did you use it:

→ I have never heard about it	1
→ I heard about it but I have never used it	2
→ I've just tried it a couple of times	3
→ I used it in the past (not over the last 30 days)	4
→ I use it occasionally (5 days or less in the last 30 days)	5
→ I use it regularly (more than 5 days in the last 30 days)	6

SECTION C. Cigarette smoking, e-cigarette use, and SHS and e-cigarette aerosol exposures in different sites

[To all]
C1. Including you, how many people live in your house? |_|_|

[To cohabitants - More than 1 at C1]
C2. How many people living in your house are aged 0-2 years? |_|_|

[To cohabitants - More than 1 at C1]
C3. How many people living in your house are aged 3-14 years? |_|_|

[To cohabitants - More than 1 at C1]
C4. Including you, how many people living in your house smoke? |_|_|

[If at least one smoker in house - More than 0 at C4]
C5. How many of them smoke inside the house
(smoking in terraces, balconies or garden should not be taken into account)? |_|_|

[To cohabitants - More than 1 at C1]
C6. And how many people use e-cigarette? |_|_|

[If at least one e-cigarette user in house - More than 0 at C6]
C7. How many of them use e-cigarettes inside the house
(smoking in terraces, balconies or garden should not be taken into account)? |_|_|

[To all]
C8. At your home, where can people (including anyone living in the household and guests) smoke?
[read the answers – only one answer)

→ Everywhere 1
→ In some specific indoor areas (e.g. in the kitchen, in the bathroom) 2
→ Nowhere inside 3
→ He/she does not know/ He/she does not answer 9

[To all]
C9. and where can they use e-cigarettes?
[read the answers – only one answer)

→ Everywhere 1
→ In some specific indoor areas (e.g. in the kitchen, in the bathroom) 2
→ Nowhere inside 3
→ He/she does not know/ He/she does not answer 9

[To all]
C10. Does your home regularly smell of smoke due to smoking from outside entering your home (e.g., neighbours or people who smoke in the vicinity, smoke from other boarding flats/houses or bars nearby, etc.)?

→ No 1
→ Yes 2
→ He/she does not know/ He/she does not answer 9

According to filters to questions C11a-d and C12 a-d, please consider the following instructions:

Questions C11a and C12a
To NON SMOKERS AND E-CIGARETTE NON-USERS
Code 1 at question B1 or B2 and code 1-4 at question B6

Questions C11b and C12b
To NON SMOKERS BUT E-CIGARETTE USERS
Code 1 at question B1 or B2 and code 5-6 at question B6

Questions C11c and C12c
To CURRENT SMOKERS BUT E-CIGARETTE NON-USERS
Code 2 at question B2 and code 1-4 at question B6

Questions C11d and C12d
To CURRENT SMOKERS AND E-CIGARETTE USERS
Code 2 at question B2 and code 5-6 at question B6

[To NON SMOKERS AND E-CIGARETTE NON-USERS]

C11a. On average, how much **time per day** do you think you are exposed to **tobacco second-hand-smoke** (SHS) and/or to **e-cigarette aerosol** in each of the following sites?

INDOOR AREAS	Exposure to tobacco smoke during an average **working day** |h|h| : |m|m|	Exposure to tobacco smoke during an average **non-working day** |h|h| : |m|m|	Exposure to e-cigarette aerosol during an average **working day** |h|h| : |m|m|	Exposure to e-cigarette aerosol during an average **non-working day** |h|h| : |m|m|
Your home	|_|_| : |_|_|	|_|_| : |_|_|	|_|_| : |_|_|	|_|_| : |_|_|
Your workplace/educational venues (for students)	|_|_| : |_|_|		|_|_| : |_|_|	
Public transportation (e.g., train, tram, bus, subway)	|_|_| : |_|_|	|_|_| : |_|_|	|_|_| : |_|_|	|_|_| : |_|_|
Private transportation	|_|_| : |_|_|	|_|_| : |_|_|	|_|_| : |_|_|	|_|_| : |_|_|
All other indoor places	|_|_| : |_|_|	|_|_| : |_|_|	|_|_| : |_|_|	|_|_| : |_|_|
Overall **[ask and check consistency with the sum]**	|_|_| : |_|_|	|_|_| : |_|_|	|_|_| : |_|_|	|_|_| : |_|_|

C12a. In the last 6 months, in your country, were people smoking traditional cigarettes and/or vaping e-cigarettes the **last time** you visited the following sites?

	never visited in the last 6 months	People smoking cigarettes		People vaping e-cigarettes	
		YES	NO	YES	NO
INDOOR (excluding smoking areas)					
A friend's or relative's home	□	□	□	□	□
A drinking establishment (such as a bar)	□	□	□	□	□
An eating establishment (such as a restaurant)	□	□	□	□	□
A disco/club/concert in an indoor arena	□	□	□	□	□
A cinema/theatre	□	□	□	□	□
A course/class in hobbies/sports	□	□	□	□	□
A public library/government office	□	□	□	□	□
An indoor train station, subway stop	□	□	□	□	□
An airport	□	□	□	□	□
A healthcare centre (e.g., hospital)	□	□	□	□	□
TRANSPORT (INDOOR)					
A car/private vehicle in presence of at least one smoker and one minor (i.e., aged<18 y)	□	□	□	□	□
A car/private vehicle in presence of at least one smoker but without minors (<18 y)	□	□	□	□	□
A public transport (tram/bus/subway)	□	□	□	□	□
A train	□	□	□	□	□
An airplane	□	□	□	□	□
OUTDOOR					
A restaurant/bar patio	□	□	□	□	□
A tram/bus/subway stop	□	□	□	□	□
An outdoor area of an hospital	□	□	□	□	□
An outdoor area of a school	□	□	□	□	□
A park	□	□	□	□	□
A children's playground	□	□	□	□	□
A stadium/outdoor arena	□	□	□	□	□
A beach	□	□	□	□	□

[To NON SMOKERS BUT E-CIGARETTE USERS]

C11b. On average, how much **time per day** do you think you are exposed to **tobacco second-hand-smoke** (SHS) and how many **e-cigarette puffs (breaths) per day** do you take in each of the following sites?

INDOOR AREAS	Exposure to tobacco SHS during an average **working day** |h|h| : |m|m|	Exposure to tobacco SHS during an average **non-working day** |h|h| : |m|m|	Use of e-cigarette during an average **working day** n° of puffs	Use of e-cigarette during an average **non-working day** n° of puffs
Your home	|_|_| : |_|_|	|_|_| : |_|_|	|_|_|_|	|_|_|_|
Your workplace/educational venues (for students)	|_|_| : |_|_|		|_|_|_|	
Public transportation (e.g., train, tram, bus, subway)	|_|_| : |_|_|	|_|_| : |_|_|	|_|_|_|	|_|_|_|
Private transportation	|_|_| : |_|_|	|_|_| : |_|_|	|_|_|_|	|_|_|_|
All other indoor places	|_|_| : |_|_|	|_|_| : |_|_|	|_|_|_|	|_|_|_|
Overall **[ask and check consistency with the sum]**	|_|_| : |_|_|	|_|_| : |_|_|	|_|_|_|	|_|_|_|

C12b. In the last 6 months, in your country, were people smoking traditional cigarettes the **last time** you visited the following sites? On that occasion, did you vape an e-cigarette?

	never visited in the last 6 months	People smoking cigarettes		Use of e-cigarette	
		YES	NO	YES	NO
INDOOR (excluding smoking areas)					
A friend’s or relative’s home	☐	☐	☐	☐	☐
A drinking establishment (such as a bar)	☐	☐	☐	☐	☐
An eating establishment (such as a restaurant)	☐	☐	☐	☐	☐
A disco/club/concert in an indoor arena	☐	☐	☐	☐	☐
A cinema/theatre	☐	☐	☐	☐	☐
A course/class in hobbies/sports	☐	☐	☐	☐	☐
A public library/government office	☐	☐	☐	☐	☐
An indoor train station, subway stop	☐	☐	☐	☐	☐
An airport	☐	☐	☐	☐	☐
A healthcare centre (e.g., hospital)	☐	☐	☐	☐	☐
TRANSPORT (INDOOR)					
A car/private vehicle in presence of at least one smoker and one minor (i.e., aged<18 y)	☐	☐	☐	☐	☐
A car/private vehicle in presence of at least one smoker but without minors (<18 y)	☐	☐	☐	☐	☐
A public transport (tram/bus/subway)	☐	☐	☐	☐	☐
A train	☐	☐	☐	☐	☐
An airplane	☐	☐	☐	☐	☐
OUTDOOR					
A restaurant/bar patio	☐	☐	☐	☐	☐
A tram/bus/subway stop	☐	☐	☐	☐	☐
An outdoor area of an hospital	☐	☐	☐	☐	☐
An outdoor area of a school	☐	☐	☐	☐	☐
A park	☐	☐	☐	☐	☐
A children’s playground	☐	☐	☐	☐	☐
A stadium/outdoor arena	☐	☐	☐	☐	☐
A beach	☐	☐	☐	☐	☐

[To CURRENT SMOKERS BUT E-CIGARETTE NON-USERS]

C11c. On average, how many **cigarettes per day** do you smoke and how much **time per day** do you think you are exposed to **e-cigarette aerosol** in each of the following sites?

INDOOR AREAS	Tobacco smoking		Exposure to e-cigarette aerosol	
	during an average **working day** n° of cigs	during an average **non-working day** n° of cigs	during an average **working day** |h|h| : |m|m|	during an average **non-working day** |h|h| : |m|m|
Your home	|_|_|	|_|_|	|_|_| : |_|_|	|_|_| : |_|_|
Your workplace/educational venues (for students)	|_|_|		|_|_| : |_|_|	
Public transportation (e.g., train, tram, bus, subway)	|_|_|	|_|_|	|_|_| : |_|_|	|_|_| : |_|_|
Private transportation	|_|_|	|_|_|	|_|_| : |_|_|	|_|_| : |_|_|
All other indoor places	|_|_|	|_|_|	|_|_| : |_|_|	|_|_| : |_|_|
Overall **[ask and check consistency with the sum]**	|_|_|	|_|_|	|_|_| : |_|_|	|_|_| : |_|_|

C12c. In the last 6 months, in your country, did you smoke a traditional cigarette the **last time** you visited the following sites? On that occasion, were people vaping e-cigarettes?

	never visited in the last 6 months	Cigarette smoking		People using e-cigarettes	
		YES	NO	YES	NO
INDOOR (excluding smoking areas)					
A friend's or relative's home	□	□	□	□	□
A drinking establishment (such as a bar)	□	□	□	□	□
An eating establishment (such as a restaurant)	□	□	□	□	□
A disco/club/concert in an indoor arena	□	□	□	□	□
A cinema/theatre	□	□	□	□	□
A course/class in hobbies/sports	□	□	□	□	□
A public library/government office	□	□	□	□	□
An indoor train station, subway stop	□	□	□	□	□
An airport	□	□	□	□	□
A healthcare centre (e.g., hospital)	□	□	□	□	□
TRANSPORT (INDOOR)					
A car/private vehicle in presence of at least one minor (i.e., aged<18 y)	□	□	□	□	□
A car/private vehicle without minors (<18 y)	□	□	□	□	□
A public transport (tram/bus/subway)	□	□	□	□	□
A train	□	□	□	□	□
An airplane	□	□	□	□	□
OUTDOOR					
A restaurant/bar patio	□	□	□	□	□
A tram/bus/subway stop	□	□	□	□	□
An outdoor area of an hospital	□	□	□	□	□
An outdoor area of a school	□	□	□	□	□
A park	□	□	□	□	□
A children's playground	□	□	□	□	□
A stadium/outdoor arena	□	□	□	□	□
A beach	□	□	□	□	□
A motorbike/scooter	□	□	□		
A bicycle	□	□	□		

[To CURRENT SMOKERS AND E-CIGARETTE USERS]

C11d. On average, how many **__cigarettes per day__** do you smoke and how many **__e-cigarette puffs (breaths) per day__** do you take in each of the following sites?

INDOOR AREAS	Tobacco smoking during an average **working day** n° of cigs	Tobacco smoking during an average **non-working day** n° of cigs	Use of e-cigarette during an average **working day** n° of puffs	Use of e-cigarette during an average **non-working day** n° of puffs
Your home	|_|_|	|_|_|	|_|_|_|	|_|_|_|
Your workplace/educational venues (for students)	|_|_|		|_|_|_|	
Public transportation (e.g., train, tram, bus, subway)	|_|_|	|_|_|	|_|_|_|	|_|_|_|
Private transportation	|_|_|	|_|_|	|_|_|_|	|_|_|_|
All other indoor places	|_|_|	|_|_|	|_|_|_|	|_|_|_|
Overall **[ask and check consistency with the sum]**	|_|_|	|_|_|	|_|_|_|	|_|_|_|

C12d. In the last 6 months, in your country, did you smoke a traditional cigarette and/or did you vape an e-cigarette the **__last time__** you visited the following sites?

	never visited in the last 6 months	Cigarette smoking		Use of e-cigarette	
		YES	NO	YES	NO
INDOOR (excluding smoking areas)					
A friend’s or relative’s home	◻	◻	◻	◻	◻
A drinking establishment (such as a bar)	◻	◻	◻	◻	◻
An eating establishment (such as a restaurant)	◻	◻	◻	◻	◻
A disco/club/concert in an indoor arena	◻	◻	◻	◻	◻
A cinema/theatre	◻	◻	◻	◻	◻
A course/class in hobbies/sports	◻	◻	◻	◻	◻
A public library/government office	◻	◻	◻	◻	◻
An indoor train station, subway stop	◻	◻	◻	◻	◻
An airport	◻	◻	◻	◻	◻
A healthcare centre (e.g., hospital)	◻	◻	◻	◻	◻
TRANSPORT (INDOOR)					
A car/private vehicle in presence of at least one minor (i.e., aged<18 y)	◻	◻	◻	◻	◻
A car/private vehicle without minors (<18 y)	◻	◻	◻	◻	◻
A public transport (tram/bus/subway)	◻	◻	◻	◻	◻
A train	◻	◻	◻	◻	◻
An airplane	◻	◻	◻	◻	◻
OUTDOOR					
A restaurant/bar patio	◻	◻	◻	◻	◻
A tram/bus/subway stop	◻	◻	◻	◻	◻
An outdoor area of an hospital	◻	◻	◻	◻	◻
An outdoor area of a school	◻	◻	◻	◻	◻
A park	◻	◻	◻	◻	◻
A children’s playground	◻	◻	◻	◻	◻
A stadium/outdoor arena	◻	◻	◻	◻	◻
A beach	◻	◻	◻	◻	◻
A motorbike/scooter	◻	◻	◻		
A bicycle	◻	◻	◻		

SECTION D. Attitudes and perceptions on smoke-free regulations and awareness of SHS harmful effects

[To all]

D1. For each of the following sites are you strongly in favour, moderately in favour, moderately against, strongly against a total tobacco ban? Moreover, are you in favour or against a ban of the use of electronic cigarettes?

	In each of the following sites, are you in favour or against a total ban of…									
	… tobacco (cigarette) smoking?					… e-cigarette use?				
SITE	Strongly **in favour**	Moderately **in favour**	Moderately **against**	Strongly **against**	Don' t know, don' t answer	Strongly **in favour**	Moderately **in favour**	Moderately **against**	Strongly **against**	Don' t know, don' t answer
INDOOR										
In private houses	□	□	□	□	□	□	□	□	□	□
Restaurants and bars	□	□	□	□	□	□	□	□	□	□
Disco/clubs/indoor arenas	□	□	□	□	□	□	□	□	□	□
Cinemas/theatres	□	□	□	□	□	□	□	□	□	□
Public libraries/government offices	□	□	□	□	□	□	□	□	□	□
Healthcare centres (including hospitals)	□	□	□	□	□	□	□	□	□	□
Schools	□	□	□	□	□	□	□	□	□	□
Universities	□	□	□	□	□	□	□	□	□	□
Train stations/subway stops	□	□	□	□	□	□	□	□	□	□
Airports	□	□	□	□	□	□	□	□	□	□
Workplaces (indoor)	□	□	□	□	□	□	□	□	□	□
TRANSPORTS (INDOOR)										
Cars/private vehicles	□	□	□	□	□	□	□	□	□	□
Cars/private vehicles with minors	□	□	□	□	□	□	□	□	□	□
Public transports (tram/bus/subway)	□	□	□	□	□	□	□	□	□	□
Trains	□	□	□	□	□	□	□	□	□	□
Airplanes	□	□	□	□	□	□	□	□	□	□
OUTDOOR										
Restaurant/bar patios	□	□	□	□	□	□	□	□	□	□
Tram/bus/subway stops	□	□	□	□	□	□	□	□	□	□
Outdoor areas of hospitals	□	□	□	□	□	□	□	□	□	□
Outdoor areas of schools	□	□	□	□	□	□	□	□	□	□
Parks	□	□	□	□	□	□	□	□	□	□
Children's playgrounds	□	□	□	□	□	□	□	□	□	□
Stadiums/outdoor arenas	□	□	□	□	□	□	□	□	□	□
Beaches	□	□	□	□	□	□	□	□	□	□
Workplaces (outdoor)	□	□	□	□	□	□	□	□	□	□

[To all]

D2. To control and limit tobacco use, the government or the national political decision-makers could adopt several strategies. How useful do you assess each one?

	Very useful	Quite useful	Rather useless	Completely useless	He/she does not know/ He/she does not answer
Free psychological or pharmacological support for smoking cessation, including nicotine replacement therapy (patches, gums, etc) bupropion and varenicline	1	2	3	4	9
Making smoking or cigarette sales illegal	1	2	3	4	9
Raising the price of cigarette	1	2	3	4	9
Extension of smoking bans	1	2	3	4	9

[To all]

D3. Do you agree or disagree with the following sentences?

	I strongly agree	I moderately agree	I moderately disagree	I strongly disagree	He/she does not know/ He/she does not answer
Exposure to SHS is harmful to my health	1	2	3	4	9
Exposure to e-cigarette vapour is harmful to my health	1	2	3	4	9
Opening the windows **in a house** where a smoker smoked could totally remove the harmful effects of SHS	1	2	3	4	9
Opening the windows **in a car** after having smoked a cigarette could totally remove the harmful effects of SHS	1	2	3	4	9
The smoke generated by a cigarette is more harmful to health than that generated by a new diesel truck	1	2	3	4	9

eTable 1. Approvals of study protocol from Ethics Committees in 12 European countries. TackSHS Project 2017-2018.

Country	Ethics Committee	Reference number	Date
Bulgaria	Ethics Committee of Bulgaria Sociological Association	-	29/06/2017
England	Imperial College Research Ethics Committee	17IC4241	21/11/2017
France	Comité de Protection des Personnes Ouest III	2017-A01860-53	9/10/2017
Germany	Ethics Committee Hamburg Medical Chamber	PV 5718	24/01/2018
Ireland	Dublin Institute of Technology	REC-17-40	20/09/2017
Greece	University of Athens	-	24/01/2018
Italy	Fondazione IRCCS Istituto Neurologico “Carlo Besta”	n. 30	8/06/2016
Latvia	Central Medical Ethics Comittee of Latvia	2/17-09-28	28/09/2017
Poland	Ethics Committee of Health Care College	1/2018	28/06/2018
Portugal	Institute of Education, University of Minho	SECVS 026/2017	31/10/2017
Romania	Comisia de Bioetica a Medicamentului si a Dispozitivelor Medicale	7SNi	7/6/2017
Spain	Bellvitge University Hospital	PR149/17	20/7/2017
